# Intrinsic Ohmic contact and electric-field tunable interface in a 2D NbS_2_/As_2_C_3_ metal–semiconductor heterostructure

**DOI:** 10.1039/d5na00949a

**Published:** 2025-12-03

**Authors:** Nguyen Xuan Sang, Nguyen Q. Cuong, Le Phuong Long

**Affiliations:** a Atomic Molecular and Optical Physics Research Group, Institute for Advanced Study in Technology, Ton Duc Thang University Ho Chi Minh City Vietnam nguyenxuansang@tdtu.edu.vn; b Faculty of Electrical and Electronics Engineering, Ton Duc Thang University Ho Chi Minh City Vietnam; c Institute of Research and Development, Duy Tan University Da Nang 550000 Vietnam nguyenquangcuong3@duytan.edu.vn; d School of Engineering & Technology, Duy Tan University Da Nang 550000 Vietnam; e Center of Scientific Research and Application, Lac Hong University No. 10 Huynh Van Nghe Str, Tran Bien Ward Dong Nai Province Vietnam phuonglong@lhu.edu.vn

## Abstract

Metal–semiconductor heterostructures play a crucial role in determining the performance of nanoelectronic and optoelectronic devices, as they govern charge injection efficiency and contact resistance at the interface. In this work, we design and explore a 2D NbS_2_/As_2_C_3_ metal–semiconductor heterostructure using first-principles calculations. The heterostructure is confirmed to be energetically stable, with weak van der Waals interactions that preserve the intrinsic characteristics of the constituent monolayers. All stacking configurations exhibit metallic behavior and form intrinsic p-type Ohmic contacts, accompanied by the absence of metal-induced gap states and only weak Fermi level pinning at the interface. The tunneling probability and contact resistivity indicate that the heterostructure exhibits low contact resistance. Furthermore, the interfacial contact behavior can be effectively tuned using an external perpendicular electric field. A positive field reduces the hole barrier while maintaining the Ohmic character, whereas a negative field enhances the barrier and induces a transition to a p-type Schottky contact. These results highlight NbS_2_/As_2_C_3_ as a promising 2D heterostructure with efficient charge injection, weak Fermi level pinning, and electric-field–tunable interfacial properties for future nanoelectronic and optoelectronic applications.

## Abstract 2

Metal–semiconductor heterostructures play a crucial role in determining the performance of nanoelectronic and optoelectronic devices, as they govern charge injection efficiency and contact resistance at the interface.

## Graphical abstract

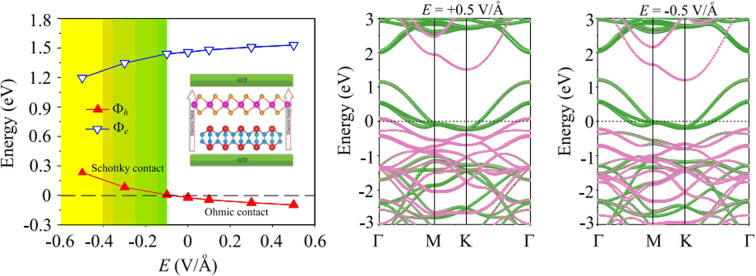

## 1 Introduction

Since the groundbreaking isolation of graphene by Novoselov and Geim in 2004,^1^ two-dimensional (2D) materials have become a rapidly expanding frontier in condensed matter physics and materials science. Graphene, a monolayer of carbon atoms arranged in a honeycomb lattice, exhibits remarkable properties such as high carrier mobility, mechanical strength, and thermal conductivity.^2,3^ However, its zero bandgap poses limitations for applications in logic devices and optoelectronics.^4^ This shortcoming has led to extensive efforts to explore alternative 2D materials with intrinsic semiconducting or magnetic properties. Among them, transition metal dichalcogenides (TMDs),^5^ hexagonal boron nitride (h-BN),^6^ black phosphorus (BP),^7^ and MXenes^8^ have emerged as promising candidates with a wide range of physical characteristics. These materials often possess tunable bandgaps, high carrier mobility, and layer-dependent phenomena, making them ideal platforms for next-generation nanoelectronics, valleytronics, and energy-related applications.^9,10^ Despite their promising properties, 2D materials face critical limitations that hinder their practical applications. For instance, although monolayer MoS_2_ has emerged as a prototypical semiconducting TMD with a direct bandgap and strong spin–orbit coupling, its carrier mobility at room temperature remains relatively low due to strong electron–phonon scattering and defects introduced during synthesis.^11,12^ In contrast, black phosphorus (BP), also known as phosphorene in its monolayer form, offers high carrier mobility and a tunable direct bandgap, making it attractive for field-effect transistors (FETs). However, BP suffers from severe ambient instability; it readily degrades upon exposure to oxygen and moisture under room temperature conditions.^13,14^ These intrinsic and extrinsic challenges necessitate strategies to stabilize or enhance the performance of 2D materials in real-world devices.

To overcome the intrinsic limitations of individual two-dimensional (2D) materials, constructing van der Waals (vdW) heterostructures has emerged as a powerful and versatile design strategy. By vertically stacking different 2D layers without the constraint of lattice matching, it becomes possible to engineer novel interfacial properties that are absent in the individual 2D materials.^15,16^ Among various configurations, metal–semiconductor heterostructures are of particular interest due to their critical roles in nanoelectronic and optoelectronic devices, including Schottky diodes, photodetectors, and field-effect transistors.^17–19^ In these systems, the interfacial band alignment and Schottky barrier height (SBH) are key factors governing charge carrier injection and overall device performance. However, in conventional metal–semiconductor contacts, large SBHs often form, limiting carrier transport. Furthermore, the Fermi level pinning (FLP) effect caused by interfacial states such as defects, metal-induced gap states (MIGSs), or strong chemical bonding can trap charges and effectively fix the Fermi level, rendering the SBH nearly insensitive to the choice of contact metal.^17^ This fundamentally restricts the tunability of the contact characteristics and often leads to high contact resistance. To address these issues, designing vdW metal–semiconductor heterostructures with weakly bonded interfaces has become an emerging direction.

Recent efforts have focused on designing vdW MSHs with weakly bonded interfaces to mitigate FLP and enhance charge transport. A wide range of such heterostructures have been successfully synthesized^20,21^ and investigated through first-principles calculations,^22–24^ encompassing integration of TMDs, graphene, MXenes, and other emerging 2D materials. These studies provide valuable insights into interfacial engineering strategies. Recently, NbS_2_, a group-V TMD, exhibits metallic conductivity even in its monolayer form, along with excellent chemical stability and strong in-plane bonding. Its high density of states near the Fermi level and good work function alignment make it a promising 2D metallic contact material for forming low-resistance interfaces with semiconducting layers.^25–29^ Furthermore, a novel 2D material, As_2_C_3_, has been computationally predicted to be a semiconductor with excellent mechanical and thermal stability^30^ that is comparable to or even superior to typical 2D semiconductors like MoS_2_ (ref. 31) and WS_2_.^32^ In its nanosheet form, As_2_C_3_ exhibits an ultrahigh carrier mobility of 4.45 × 10^5^ cm^2^ V^−1^ s^−1^, which is comparable to that of graphene^33^ and even larger than those of other common 2D semiconductors such as MoS_2_ (ref. 34) and phosphorene.^35^ In addition, As_2_C_3_ has been proposed as a promising candidate for gas sensing applications,^36^ as well as for integration with a variety of 2D materials, including both metallic and semiconducting systems.^37–39^ These findings strongly suggest that employing 2D As_2_C_3_ as a channel material could open new opportunities for the design of next-generation nanodevices.

In this work, we computationally design and investigate the integration of 2D metallic NbS_2_ and semiconducting As_2_C_3_ to form a novel van der Waals heterostructure. We systematically explore its electronic properties and interfacial contact engineering, with particular emphasis on the effects of external electric fields. Our results reveal the fundamental mechanisms governing charge transfer, band alignment, and contact formation, thereby providing valuable insights into interface engineering strategies for next-generation 2D nanoelectronic devices based on the metal–semiconductor NbS_2_/As_2_C_3_ heterostructure.

## 2 Computational details

First-principles calculations based on density functional theory (DFT) were carried out using the Vienna *Ab initio* Simulation Package (VASP).^40^ The projector augmented wave (PAW) method was employed to describe the interaction between core and valence electrons.^41^ Electron exchange and correlation were treated using the Perdew–Burke–Ernzerhof (PBE) functional^42,43^ within the generalized gradient approximation (GGA).^44^ To account for the weak interlayer van der Waals (vdW) interactions crucial for heterostructure modeling, the DFT-D3 method with Becke–Johnson damping was applied.^45^ A plane-wave basis set with an energy cutoff of 500 eV was used throughout all simulations. For Brillouin zone sampling, a Monkhorst–Pack *k*-point grid of 12 × 12 × 1 was employed. These parameters are also consistent with previous DFT studies.^46–48^ All atomic structures were fully relaxed until the residual forces on each atom were less than 0.01 eV Å^−1^, and the total energy convergence threshold was set to 10^−6^ eV. A vacuum region of 20 Å was included along the out-of-plane direction to avoid spurious interactions between periodic images.

## 3 Results and discussion

**Fig. 1:**
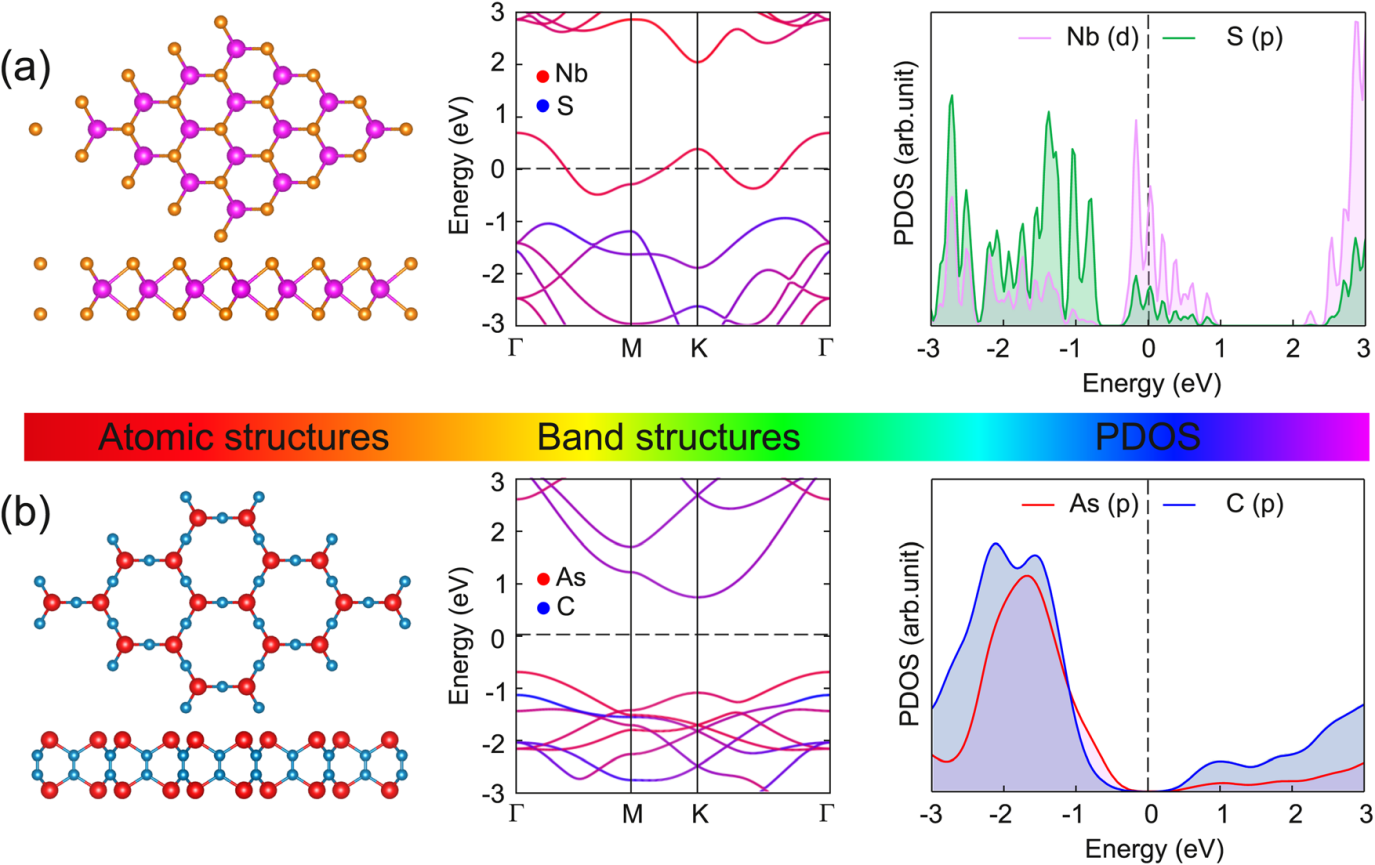
Atomic structures, projected band structures and density of states of (a) NbS_2_ and (b) As_2_C_3_ monolayers. Orange and purple circles denote the S and Nb atoms, respectively, while red and blue circles stand for the As and C atoms, respectively.

We begin our investigation by examining the atomic and electronic structures of the individual monolayers of NbS_2_ and As_2_C_3_, as shown in Fig. 1. The NbS_2_ monolayer adopts a layered hexagonal structure belonging to the *P*6_3_/*mmc* space group and possesses D 3 h point group symmetry. Its unit cell comprises a single niobium (Nb) atom coordinated by two sulfur (S) atoms, forming a sandwich-like configuration with trigonal prismatic coordination. The optimized lattice constant is found to be 3.41 Å, in good agreement with previous theoretical studies.^49^ The electronic band structure of NbS_2_ reveals a metallic character, characterized by a conduction band that intersects the Fermi level. This metallic behavior arises primarily from the strong hybridization between Nb-d orbitals and S-p orbitals, as clearly illustrated in the projected density of states (PDOS) in Fig. 1(a). The presence of a high density of states near the Fermi energy further confirms the suitability of NbS_2_ as a 2D metallic contact material for heterostructure applications. The atomic structure of the As_2_C_3_ monolayer is illustrated in Fig. 1(b). Similar to NbS_2_, As_2_C_3_ adopts a layered hexagonal configuration and crystallizes in the *P*6/*mmm* space group. Its unit cell comprises four arsenic (As) atoms and six carbon (C) atoms, forming a stable 2D framework. The optimized lattice constant is calculated to be 5.86 Å, which is consistent with previous theoretical predictions.^30,36^ At the ground state, monolayer As_2_C_3_ exhibits an indirect semiconducting nature with a band gap of 1.43 eV within the PBE functional and 2.31 eV when computed using the HSE06 hybrid functional. The valence band maximum (VBM) is primarily derived from As-p orbitals, while the conduction band minimum (CBM) arises from the hybridization between As-p and C-p states. The sizable band gap indicates that As_2_C_3_ is a promising candidate for semiconducting components in 2D heterostructures.

**Fig. 2:**
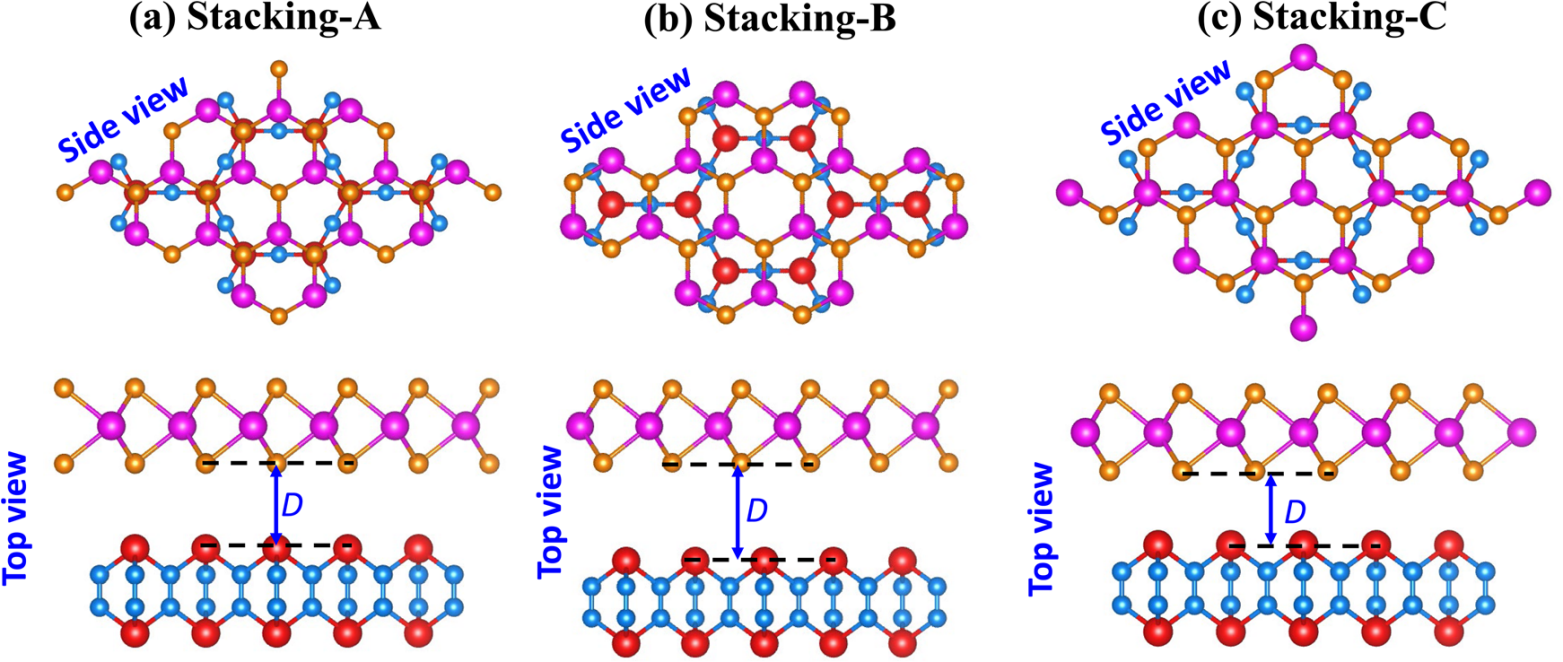
Optimized atomic structures of the NbS_2_/As_2_C_3_ heterostructure for different stacking configurations of (a) Stacking-A, (b) Stacking-B and (c) Stacking-C.

To explore the interfacial properties of the 2D NbS_2_/As_2_C_3_ heterostructure, we constructed vertically stacked models by aligning the constituent monolayers along the out-of-plane direction. Owing to the intrinsic symmetry and layered characteristics of both NbS_2_ and As_2_C_3_, three distinct stacking configurations were proposed and systematically investigated, as illustrated in Fig. 2. These configurations are referred to as Stacking-A, Stacking-B, and Stacking-C. Each stacking arrangement was fully relaxed to obtain the energetically favorable atomic structure prior to electronic property analysis. The equilibrium interlayer separations between the NbS_2_ and As_2_C_3_ layers in each heterostructure, denoted as D, are highlighted in Fig. 2. The obtained values of D for Stacking-A, Stacking-B and Stacking-C are 3.50, 3.61 and 3.20 Å, respectively. Among them, Stacking-C exhibits the shortest interlayer distance, suggesting a relatively stronger interlayer interaction. Additionally, as compared with the interlayer separations in other typical vdW heterostructures, such as M, the interlayer separations D obtained for the NbS_2_/As_2_C_3_ heterostructures fall well within the typical vdW interaction range. This observation confirms that the interlayer coupling in the proposed heterostructures is governed by vdW forces. Furthermore, to assess the thermodynamic stability of the proposed NbS_2_/As_2_C_3_ heterostructures, we evaluate the binding energy (*E*_b_) for each stacking configuration. The binding energy is defined as1
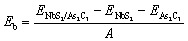


Here, the total energies of the constituent NbS_2_, As_2_C_3_ and their heterostructure are denoted by *E*_NbS_2__, *E*_As_2_C_3__ and *E*_NbS_2_/As_2_C_3__, respectively. *A* stands for the area of the considered supercell for the heterostructure. The calculated binding energies per unit area for Stacking-A, Stacking-B, and Stacking-C are −12.73, −11.80 and −20.30 meV Å^−2^, respectively. All values are negative, indicating that the formation of the NbS_2_/As_2_C_3_ heterostructure is energetically favorable in all three configurations. Among them, Stacking-C exhibits the most negative binding energy, suggesting that it is the most energetically stable configuration. This is consistent with the fact that Stacking-C also possesses the shortest interlayer spacing (3.20 Å), reflecting the weak vdW interaction between the two layers. Importantly, the interaction remains in the vdW regime, which does not alter the intrinsic electronic properties of the constituent monolayers but merely holds them together, similar to the behavior observed in graphite and many other 2D vdW heterostructures. Consequently, in 2D heterostructures, the most stable stacking configurations—those exhibiting the lowest (most negative) binding energies—typically correspond to the smallest interlayer separations. These trends have also been consistently reported in various other 2D systems, further supporting the reliability of our observations.^50–52^ Additionally, these values of the binding energy are comparable with those obtained in graphite^53^ and other vdW heterostructures.^54,55^

**Fig. 3:**
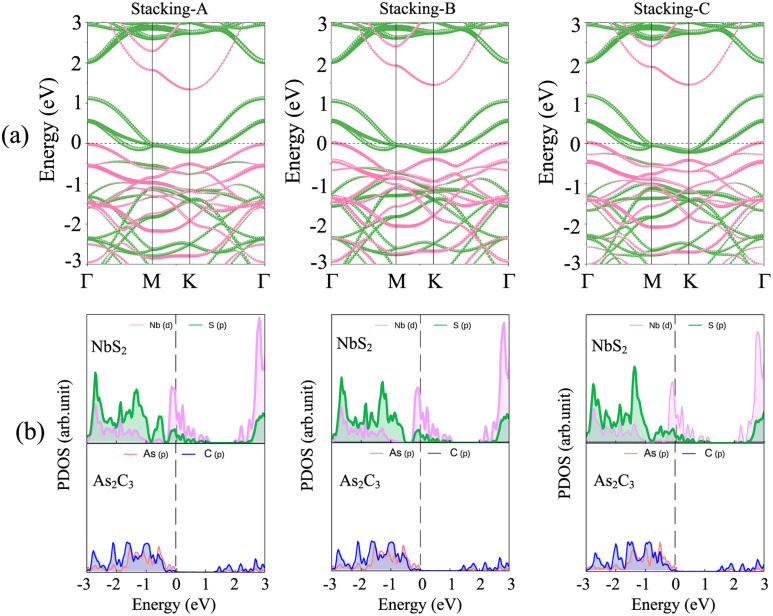
(a) PBE projected band structures, (b) density of states and (c) HSE06 projected band structures of the NbS_2_/As_2_C_3_ heterostructure for different stacking configurations. Green and pink lines represent the contributions of the NbS_2_ and As_2_C_3_ layers, respectively.

We further investigate the electronic characteristics of the NbS_2_/As_2_C_3_ heterostructure by analyzing its electronic band structures and projected density of states (PDOS), as presented in Fig. 3. Remarkably, all three stacking configurations retain a metallic nature, with bands crossing the Fermi level. This observation indicates that the metallic character of the NbS_2_ layer is preserved upon stacking with the semiconducting As_2_C_3_ monolayer, as displayed in Fig. 3(a). The metallicity in the heterostructure is mainly governed by the hybridization between Nb-d and S-p orbitals, as evidenced by the dominant contribution near the Fermi level in the PDOS in Fig. 3(b). The preservation of metallic states is of particular interest for applications in electrical contacts, as it suggests efficient carrier injection. More interestingly, we can observe that in all three stacking configurations, the VBM of the As_2_C_3_ layer crosses the Fermi level at the *Γ* point. This characteristic implies that hole carriers from the semiconducting As_2_C_3_ layer can be injected into the metallic NbS_2_ layer without encountering an energy barrier. Consequently, Ohmic contact is established at the interface for all three configurations. The formation of Ohmic contact is highly desirable for nanoelectronic applications, as it ensures minimal contact resistance and efficient charge transport across the interface. Interestingly, the PDOS analysis reveals that there are no significant electronic states within the band gap region of the As_2_C_3_ layer contributed by the metallic NbS_2_ side. This absence of metal-induced gap states (MIGSs) suggests that the interlayer interaction at the NbS_2_/As_2_C_3_ interface is predominantly governed by vdW forces. Consequently, the Fermi level remains unpinned and aligns closely with the valence band maximum of the semiconductor. This weak FLP behavior enhances the flexibility of designing low-resistance electrical contacts in 2D devices and further underscores the potential of the NbS_2_/As_2_C_3_ heterostructure for application in future nanoelectronic and optoelectronic devices.

**Fig. 4:**
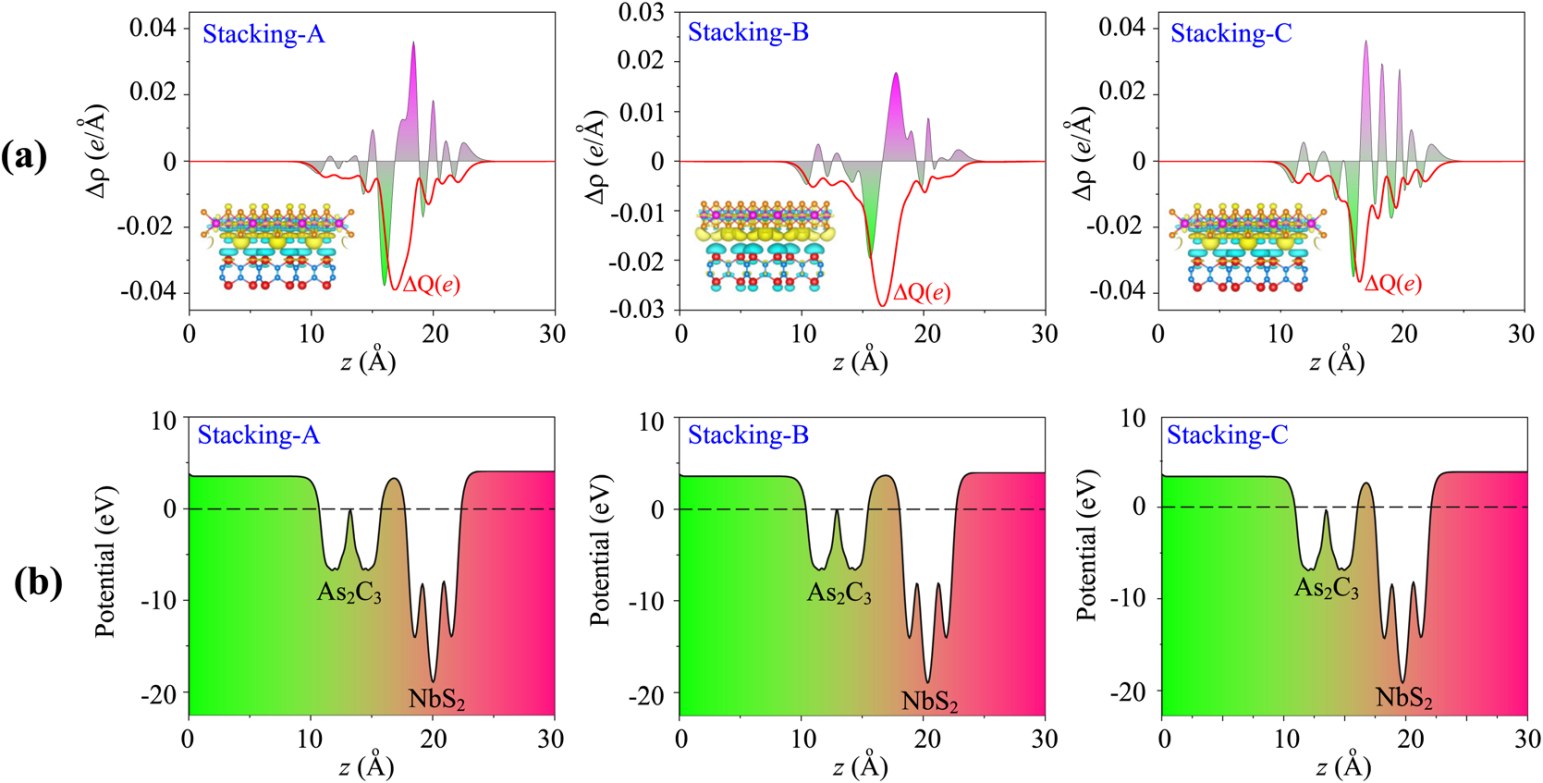
(a) Charge density difference and (b) electrostatic potential of the NbS_2_/As_2_C_3_ heterostructure for different stacking configurations. The insets in Fig. 4(a) show the 3D isosurfaces. The charge accumulation and depletion are represented by the yellow and cyan colors, respectively.

To gain deeper insight into the interfacial interactions, we further examine the charge redistribution at the interface of the NbS_2_/As_2_C_3_ heterostructures, as illustrated in Fig. 4(a). The charge density difference is defined as follows:

2 Δ*ρ*(***r***) = *ρ*_NbS_2_/As_2_C_3__(***r***) − *ρ*_NbS_2__(***r***) − *ρ*_As_2_C_3__(***r***)

Here, *ρ*_NbS_2_/As_2_C_3__(***r***), *ρ*_NbS_2__(***r***) and *ρ*_As_2_C_3__(**r**) are the charge densities of the NbS_2_/As_2_C_3_ heterostructure and NbS_2_ and As_2_C_3_ monolayers, respectively. As illustrated in Fig. 4, the yellow regions, indicating charge accumulation, are primarily located on the NbS_2_ side, whereas the cyan regions, corresponding to charge depletion, are found near the As_2_C_3_ layer. This spatial distribution suggests that electrons are transferred from the As_2_C_3_ semiconductor to the metallic NbS_2_ layer upon the formation of the heterostructure. Such a charge transfer results in the formation of an interfacial dipole directed from the semiconductor to the metal, which slightly modifies the local potential at the interface. Furthermore, to obtain the total amount of charge transfer from the As_2_C_3_ to the NbS_2_ layers, we calculate the cumulative charge density along the *z* direction as follows:3
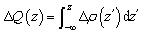
The values of Δ*Q* for the NbS_2_/As_2_C_3_ heterostructure in all three stacking configurations are illustrated in Fig. 4(a). It can be observed that the amount of charge transferred across the interface lies in the range of 0.03 to 0.04 electrons. In addition, an atomic population analysis based on the Bader charge scheme^56^ was performed to quantify the charge transfer between the NbS_2_ and As_2_C_3_ layers. The Bader charge population analysis further confirms that a small amount of charge of 0.035 ÷ 0.042 e is transferred from As_2_C_3_ to NbS_2_ layers upon heterostructure formation, which agrees well with the planar-integrated charge density difference Δ*Q* ≈ 0.03 ÷ 0.04 e. These relatively small values indicate that the interlayer interaction is dominated by weak van der Waals (vdW) forces. This minimal charge redistribution also implies a weak electronic coupling between the two layers, which is consistent with the absence of metal-induced gap states (MIGSs) and the weak Fermi level pinning (FLP) observed in the projected density of states (PDOS).

Furthermore, the electrostatic potential profiles of the NbS_2_/As_2_C_3_ heterostructures are plotted in Fig. 4(b). It is evident that the potential well of the NbS_2_ layer is deeper than that of the As_2_C_3_ layer across all three stacking configurations. This indicates an intrinsic electrostatic field within the NbS_2_ monolayer. Moreover, the calculated work functions reveal that the As_2_C_3_ side possesses a lower work function compared to the NbS_2_ side. This difference in work function drives a small electron transfer from As_2_C_3_ to NbS_2_, consistent with the observed Δ*Q* values and the direction of the built-in potential gradient. Additionally, analysis of the electrostatic potential across the NbS_2_/As_2_C_3_ heterostructure enables the extraction of critical interface parameters, such as the contact barrier height (*Φ*_B_) and barrier width (*W*_B_). These quantities are essential for evaluating charge injection efficiency at the metal–semiconductor junction. Based on the extracted *Φ*_B_ and *W*_B_, we estimate the tunneling probability (*T*_B_) using the Wentzel–Kramers–Brillouin (WKB) approximation, which provides insight into the likelihood of carrier transmission across the interface. Furthermore, the contact resistivity (*ρ*_t_) can be derived from the tunneling probability, offering a quantitative measure of the interfacial transport characteristics. The *T*_B_ and *ρ*_t_ can be quantitatively estimated using the following expressions:4
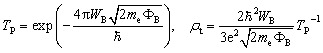
The calculated Schottky barrier width (*W*_B_) and height (*Φ*_B_) of the NbS_2_/As_2_C_3_ heterostructure are found to be 3.28 eV and 1.90 Å for Stacking-A, 3.64 eV and 2.57 Å for Stacking-B, and 2.83 eV and 1.40 Å for Stacking-C, respectively. Based on eqn (4), the corresponding tunneling probability (*T*_B_) and contact resistivity (*ρ*_t_) are further evaluated to be 2.94% and 7.53 × 10^−10^ Ωcm^2^ for Stacking-A, 0.66% and 4.32 × 10^−9^ Ωcm^2^ for Stacking-B, and 8.95% and 1.96 × 10^−10^ Ωcm^2^ for Stacking-C. The obtained *T*_B_ values indicate that electrons can effectively tunnel through the relatively narrow Schottky barriers, especially in the case of Stacking-C, where the smallest barrier width facilitates the highest tunneling probability. Consequently, the contact resistivity *ρ*_t_ is predicted to be the lowest for Stacking-C, highlighting its potential as the most favorable configuration for efficient charge injection in NbS_2_/As_2_C_3_ heterostructure-based nanoelectronic devices.

**Fig. 5:**
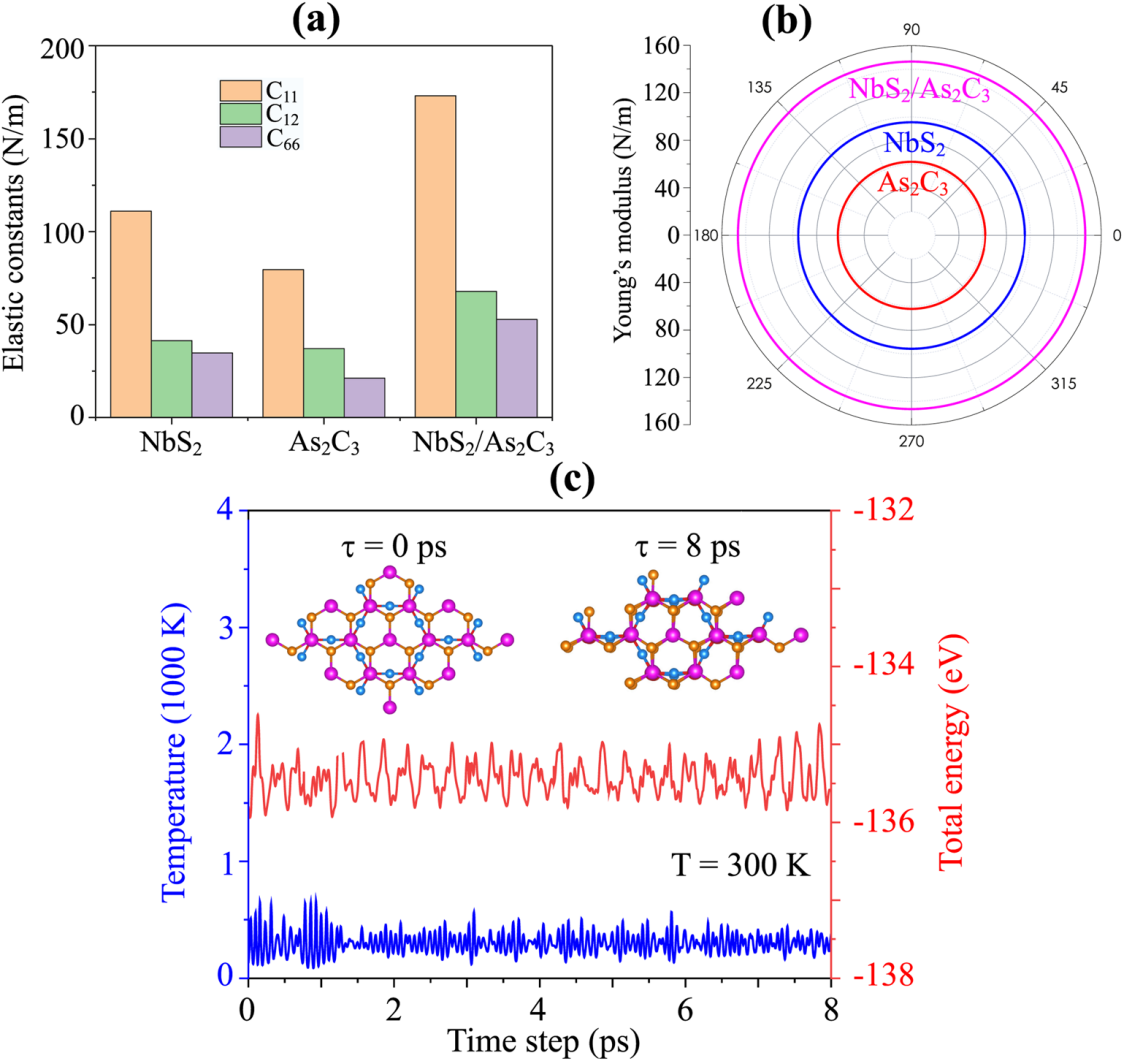
(a) In-plane elastic constants and (b) Young's modulus of the NbS_2_/As_2_C_3_ heterostructure and the constituent NbS_2_ and As_2_C_3_ monolayers. (c) AIMD simulation of the total energy and temperature of the Stacking-C of the NbS_2_/As_2_C_3_ heterostructure. The insets show the atomic structures before and after simulation.

As discussed above, Stacking-C of the NbS_2_/As_2_C_3_ heterostructure is identified as the most energetically favorable configuration. To further assess its suitability for practical applications, we evaluate its mechanical and thermal stability. Mechanical stability is examined through the calculation of elastic constants and Young's modulus. Thermal stability is investigated *via ab initio* molecular dynamics (AIMD) simulations performed at room temperature *T* = 300 K over an 8 ps timescale. It is evident that the formation of the NbS_2_/As_2_C_3_ heterostructure induces a notable enhancement in the elastic constants compared to those of the individual monolayers, as illustrated in Fig. 5(a). The calculated in-plane elastic constants for the NbS_2_/As_2_C_3_ heterostructure are C_11_ = 173.02 N m^−1^, C_12_ = 67.77 N m^−1^ and C_44_ = 52.62 N m^−1^. One can find that these values of the elastic constants satisfy the Born-Huang mechanical stability criteria for 2D materials, which require that C_11_ > 0, C_44_ > 0 and C_11_ − C_12_ > 0.^31,57,58^ The fulfillment of these conditions confirms that the NbS_2_/As_2_C_3_ heterostructure is mechanically stable under small deformations. Furthermore, we calculated the angular dependence of Young's modulus of the NbS_2_/As_2_C_3_ heterostructure to further assess its mechanical rigidity as follows:^59,60^5
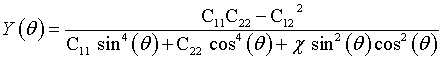
where 
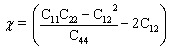
. The polar dependence of the Young's modulus for the NbS_2_/As_2_C_3_ heterostructure is illustrated in Fig. 5(b). The heterostructure exhibits isotropic mechanical behavior in the plane. The Young's modulus reaches a maximum value of 146.47 N m^−1^, which is higher than that of the constituent monolayers. In addition, this value is comparable with that in other 2D materials, such as MoS_2_ (130 N m^−1^)^61^ and Ti_2_C MXene (130 N m^−1^).^62^ This isotropy suggests that the mechanical response of the heterostructure is uniform regardless of the direction of applied strain, a desirable property for flexible and mechanically stable 2D devices. Furthermore, the *ab initio* molecular dynamics (AIMD) simulation results for the NbS_2_/As_2_C_3_ heterostructure are presented in Fig. 5(c). Throughout the simulation, no structural distortions, bond breaking, or significant atomic displacements were observed, confirming the thermal robustness of the heterostructure under elevated temperature conditions. All these findings indicate that the NbS_2_/As_2_C_3_ heterostructure demonstrates both mechanical integrity and thermal resilience, reinforcing its promise for reliable integration into next-generation 2D nanoelectronic and optoelectronic devices.

**Fig. 6:**
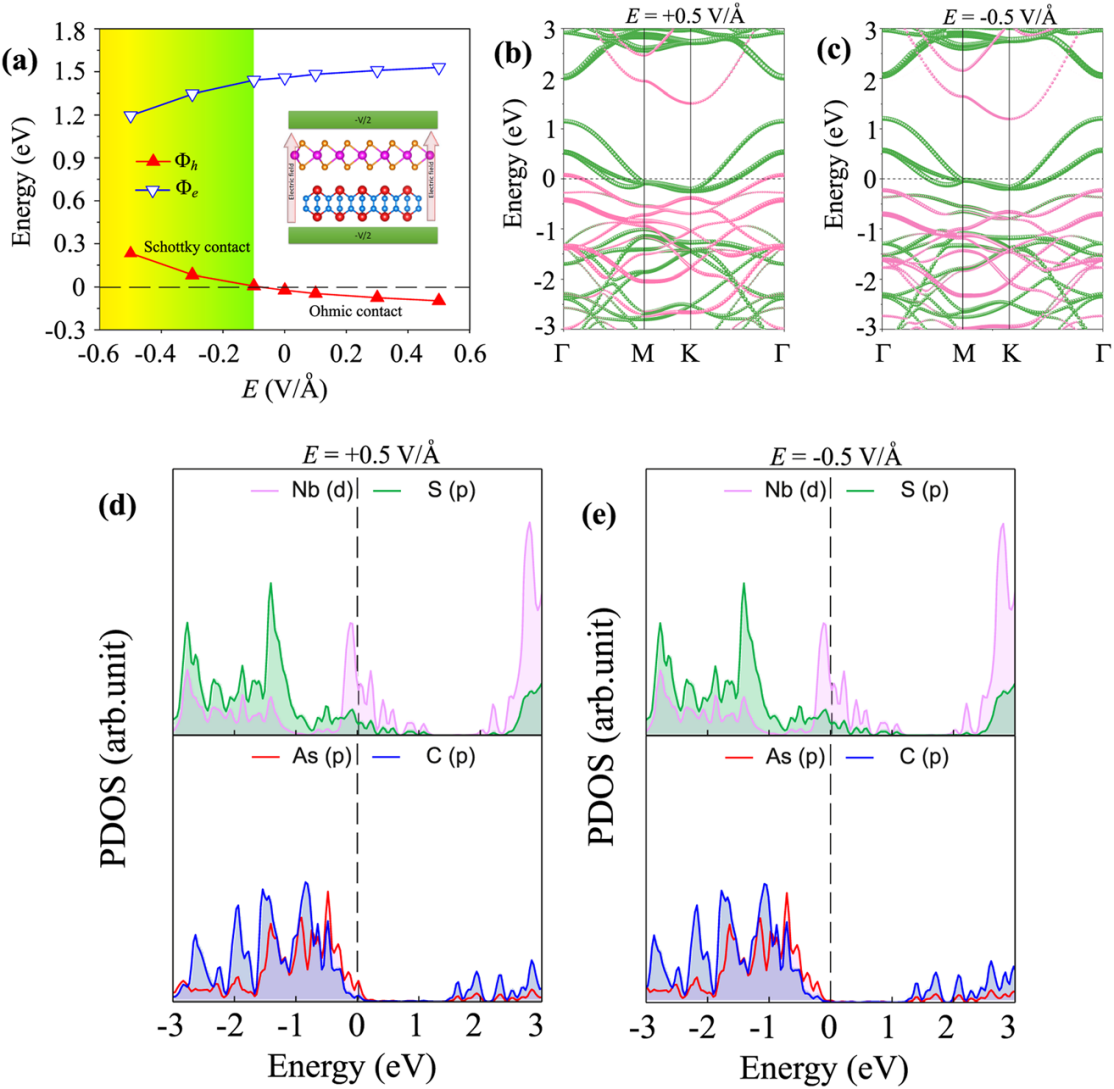
(a) The fluctuation of the Schottky barriers of the NbS_2_/As_2_C_3_ heterostructure under applied electric fields. The inset shows the schematic illustration of the electric field along the *z* direction. The projected band structures of the NbS_2_/As_2_C_3_ heterostructure under (b) positive *E* = +0.5 V Å^−1^ and (c) negative *E* = −0.5 V Å^−1^. The PDOS for all atoms in the NbS_2_/As_2_C_3_ heterostructure under (d) positive *E* = +0.5 V Å^−1^ and (e) negative *E* = −0.5 V Å^−1^.

Furthermore, the practical implementation of the metal–semiconductor NbS_2_/As_2_C_3_ heterostructure hinges on its tunability in electronic properties and contact performance, which are critical for optimizing device functionality. To this end, we investigate whether these characteristics can be modulated through external stimuli, such as an applied perpendicular electric field. This approach enables a deeper understanding of the field-induced effects on band alignment and Schottky barriers, thereby assessing the feasibility of tailoring the heterostructure electronic behavior for specific nanoelectronic applications. The direction of the applied external electric field is illustrated in the inset of Fig. 6(a). A positive electric field is defined as pointing from the As_2_C_3_ layer toward the NbS_2_ layer, *i.e.*, perpendicular to the interface and aligned with the out-of-plane axis and its strength varies from −0.5 to +0.5 V Å^−1^. Such large strengths of applied electric fields can be generated in recent experiments using dual ion-in-liquid gating.^63^ The application of an external electric field introduces tunability in both the contact barrier heights and the contact types of the NbS_2_/As_2_C_3_ heterostructure. Under a positive electric field, the hole barrier is significantly reduced, while the electron barrier increases correspondingly. This asymmetric modulation preserves the p-type Ohmic contact nature of the heterostructure, facilitating efficient hole injection from NbS_2_ into As_2_C_3_. Conversely, the application of a negative electric field leads to an increase in the hole barrier and a reduction in the electron barrier. Notably, when the field strength exceeds −0.2 V Å^−1^, the hole barrier becomes significantly enhanced and attains a positive value, indicating the emergence of a p-type Schottky contact at the NbS_2_/As_2_C_3_ interface. The tunability in the contact types and contact barriers of the NbS_2_/As_2_C_3_ heterostructure makes it a promising candidate for next-generation nanoelectronic and optoelectronic devices. The projected band structures of the NbS_2_/As_2_C_3_ heterostructure under external electric fields of +0.5 V Å^−1^ and −0.5 V Å^−1^ are shown in Fig. 6(b) and (c), respectively. Under the application of a positive electric field of +0.5 V Å^−1^, the heterostructure retains its Ohmic contact character, as evidenced by the presence of bands crossing the Fermi level. These crossing bands indicate efficient carrier injection and minimal energy barrier at the interface. The states near the Fermi level arise from the hybridization between the metallic NbS_2_ layer and the As-p orbitals of the As_2_C_3_ layer, as illustrated in the PDOS of the NbS_2_/As_2_C_3_ heterostructure in Fig. 6(d). More interestingly, under the application of a negative electric field of −0.5 V Å^−1^, as shown in Fig. 6(c), the NbS_2_/As_2_C_3_ heterostructure undergoes a transition from Ohmic to Schottky contact. This shift is characterized by a downshift in the valence band of the As_2_C_3_ layer toward lower binding energies, effectively increasing the hole injection barrier and reinforcing the Schottky contact nature. Under the negative electric field, the bands crossing the Fermi level are contributed by the metallic NbS_2_ layer with no contribution from the As_2_C_3_ layer, as confirmed by the projected density of states (PDOS) shown in Fig. 6(e). All these findings suggest that the electric field serves as an effective external stimulus to modulate the electronic properties and contact behavior of the NbS_2_/As_2_C_3_ heterostructure. This tunability highlights the potential of such NbS_2_/As_2_C_3_ heterostructures as promising candidates for next-generation field-effect transistors and nanoelectronic devices.

## 4 Conclusions

In summary, we have proposed and investigated a novel 2D NbS_2_/As_2_C_3_ metal–semiconductor heterostructure using first-principles calculations. The heterostructure is found to be energetically stable with weak interlayer interactions that preserve the intrinsic properties of the constituent layers. All stacking configurations exhibit metallic features, and importantly, the system forms intrinsic p-type Ohmic contacts owing to the crossing of the As_2_C_3_ valence band through the Fermi level. The projected density of states further reveals the absence of metal-induced gap states and only weak Fermi level pinning at the interface. The calculated tunneling probability and contact resistivity indicate that the Stacking-C configuration is the most favorable, offering the lowest resistance due to its narrowest barrier width. Charge transfer analysis also confirms electron transfer from As_2_C_3_ to NbS_2_, consistent with the interfacial band alignment. Furthermore, the application of a perpendicular electric field is shown to effectively tune the interfacial properties. A positive field reduces the hole barrier while preserving the Ohmic character, whereas a negative field enhances the hole barrier and induces a transition to a p-type Schottky contact. These findings demonstrate that NbS_2_/As_2_C_3_ is a promising 2D metal–semiconductor heterostructure with efficient charge injection, weak Fermi level pinning, and tunable contact properties. This work provides valuable insights into interfacial engineering strategies and paves the way for the design of next-generation nanoelectronic and optoelectronic devices based on emerging 2D materials.

## Conflicts of interest

There are no conflicts to declare.

## Data Availability

The data that support the findings of this study are available from the corresponding author upon reasonable request.
